# Termite usage associated with antibiotic therapy: enhancement of aminoglycoside antibiotic activity by natural products of *Nasutitermes corniger *(Motschulsky 1855)

**DOI:** 10.1186/1472-6882-9-35

**Published:** 2009-09-17

**Authors:** Henrique DM Coutinho, Alexandre Vasconcellos, Micheline A Lima, Geraldo G Almeida-Filho, Rômulo RN Alves

**Affiliations:** 1 Universidade Regional do Cariri, Laboratório de Microbiologia e Biologia Molecular, Crato (CE), 63105-000, Brazil; 2 Universidade Federal do Rio Grande do Norte, Departamento de Botânica, Ecologia e Zoologia, Natal (RN), 59072-900, Brazil; 3 Universidade Federal de Campina Grande, Unidade Acadêmica de Saúde, Cuité (PB) Brazil; 4 Universidade Federal da Paraíba - UFPB; Departamento de Biologia Molecular - DBM, 58051-900, Brazil; 5 Departamento de Biologia, Universidade Estadual da Paraíba, Campina Grande, Paraíba (PB), 58109-753, Brazil

## Abstract

**Background:**

Several species from Insecta are used as remedies. Among these species, the termite *Nasutitermes corniger *is commonly used in traditional medicine in Northeast Brazil. The present work tests the modifying antibiotic activity of *Nasutitermes corniger*, a termite used in folk medicine in Northeastern region of Brazil.

**Methods:**

Chlorpromazine and decocts of *N. corniger *were collected from two different plant species used in the traditional medicine were tested for their antimicrobial activity against strains of *Escherichia coli *resistant to aminoglycosides. The growth of two bacterial strains of *E. coli *was tested using decocts and chlorpromazine alone or associeted with aminogycosides.

**Results:**

The MIC and MBC values were ≥1024 μg/ml for both strains of *E. coli *assayed. A significant synergism was observed between both decocts and chlorpromazine when assyed with neomycin. This synergism with neomycin indicates the involvement of an efflux system in the resistance to this aminoglycoside.

**Conclusion:**

Therefore it is suggested that natural products from *N. corniger *could be used as a source of zoo-derived natural products with modifying antibiotic activity to aminoglycosides, being a new weapon against the bacterial resistance to antibiotics.

## Background

Although medicinal plants are well studied around the world, animals or animal parts have been broadly used in Brazilian traditional medicine and have played a significant role in healing practices [1,2]. Several species from Insecta have been used as remedies [1-4]. Among these species, the Neotropical termite *Nasutitermes corniger *is commonly used in traditional medicine in Northeast Brazil. *N. corniger *is distributed from southern Mexico to northern Argentina and the West Indies and is spread from the semi-arid to tropical rain forest ecosystems [5-9]. In South America, this species is highly adaptable to colonizing contrasting habitats in urban, agricultural, and natural environments [10,11]. *N. corniger *builds arboreal carton nests with a population that can exceed 400,000 individuals/nest, and density that ranges from 22.1 to 47.1 nest/ha in tropical rain forests [10,12,13]. Based on morphologic, genetic and biogeographic evidence, *N. costalis *has been revised as a synonym of *N. corniger*, like the congeneric species *N. araujoi*, *N. globiceps *and *N. tatarendae *which are also synonyms of *N. corniger *[8,9].

With the increase in microbial resistance to antibiotics, the use of natural products represent an interesting alternative for treatment [14,15]. Many products have been evaluated not only for direct antimicrobial activity, but also as resistance modifying agents [16,17]. Several chemical compounds, from synthetic or biological sources, such as phenothiazines and natural products, have direct activity against many bacteria, enhancing the activity of a specific antibiotic, reversing the natural resistance of specific bacteria to several antibiotics, promoting the elimination of plasmids from bacteria such as *Escherichia coli*, and inhibiting drug-transport functions of the plasma membrane. Inhibition of plasma membrane-based efflux pumps has been observed as well [18,19]. The enhancement of antibiotic activity or the reversal of antibiotic resistance by natural or synthetic non-conventional antibiotics results in the classification of these compounds as modifiers of antibiotic activity. Aminoglycosides are potent bactericidal antibiotics that target the bacterial ribosome and development of bacterial resistance to aminoglycosides is widely recognized as a serious health threat [18]. In *E. coli*, the main mechanisms of resistance to aminoglycosides are active drug efflux and enzymatic inactivation [19].

The aim of the present study was to evaluate the decoctions of *N. corniger *obtained from two different trees *(Commiphora leptophloeos Mart J. B. Gillet *and *Anacardium occidentale L.) *as resistance-modifying agents against *E. coli*.

## Methods

### Strains

The strain used was the clinical isolate *Escherichia coli *(EC27), resistant to neomycin and gentamicin (low level) and to amikacin and kanamycin. The strain *Escherichia coli *(EC - ATCC8539) was used as the positive control. All strains were maintained in heart infusion agar slants (HIA, Difco), and prior to assay, the cells were grown overnight at 37°C in brain heart infusion (BHI, Difco).

### Zoological and plant material

*Nasutitermes corniger *was collected in the county of Alagoa Nova, Paraíba, Brazil (21°58'N, 89°36'W) during the month of June 2007. The samples were authenticated by Prof. Alexandre Vasconcellos at the Botany, Ecology and Zoology Department, UFRN. A voucher specimen (CICB 68 and CICB 69) was deposited in the Isoptera Collection of the Bioscience Center, Universidade Federal do Rio Grande do Norte - UFRN. The botanical identification of the plants was performed at the "Herbarium Prof. Lauro Pires Xavier" (JPB), Department of Systematics and Ecology, in the Universidade Federal da Paraíba, Brazil, where voucher specimens were preserved under the reference numbers JPB 37775 for *Commiphora leptophloeos *and JPB 37745 for *Anacardium occidentale*.

### Preparation of *N. corniger *decoctions from *Commiphora leptophloeos *(DCL) and *Anacardium occidentale *(DAO)

Two hundred grams of termites with nests were collected and powdered. The powdered material was extracted by maceration using 100 mL of sterile water as solvent at room temperature. The mixture was allowed to stand for 72 h at room temperature. Decoctions were then filtered and assayed to determine antibacterial activity.

### Drugs

Chlorpromazine, gentamicin, kanamycin, amikacin and neomycin were obtained from SIGMA. All drugs were dissolved in sterile water.

### Drug susceptibility test and determination of fractional inhibitory concentration (FIC)

The minimum inhibitory concentrations (MICs) of the decoctions, antibiotics and chlorpromazine (CPZ) were determined in BHI by the microdilution assay using suspensions of 10^5 ^cfu/mL and a drug concentration range of 1024 to 1 μg/mL (two-fold serial dilutions) [20]. The MIC was defined as the lowest concentration at which no growth was observed. For the evaluation of the decoctions as a modulator of antibiotic resistance, the MICs of the antibiotics were determined in the presence of the decoctions and CPZ at a sub-inhibitory concentration and the FIC calculated. The fractional inhibitory concentration (FIC) was used to interpret the dilution method results and was calculated as follows [21]: FIC of drug A = MIC drug A in combination with decoction or CPZ/MIC drug A alone. Synergy was defined as FIC < 0.5; indifference was defined as 4 > FIC > 0.5; and antagonism was defined as an FIC > 4. The plates were incubated for 24 h at 37°C. CPZ was used as the positive control for efflux pump modulation, due to the fact it affects the function of efflux pumps. All experiments were realized in duplicate.

## Results

Both decoctions showed no substantial antibacterial activity at 1024 μg/mL against the strains tested (MIC ≥ 2048 μg/mL). However, when the decoction of termites from *C. leptophloeos *was added to the growth medium at 256 μg/mL (≥1/8 MIC), a reduction of the MIC for gentamicin and neomycin was observed in the strain *E. coli *27 (but not in ATCC 8539), demonstrating a synergistic or additive effect of this natural product with these aminoglycosides (Table 1). The decoction of termites from *A. occidentale *L. shown synergism only against neomycin (Table 2), demonstrating the influence of the plant substrate in the pharmacological properties of this natural product of termites.

**Table 1:** Evaluation of the Modifying Antibiotic Activity of the Decoct of insects from *Commiphora leptophloeos *(256 μg/mL) and CPZ (16 μg/mL) against aminoglycosides.

	**EC 27**			**EC ATCC8539**		
	**MIC**	**MIC combined**		**MIC**	**MIC combined**	

Antibiotics	-	DCL/FIC	CPZ/FIC	-	DCL/FIC	CPZ/FIC

Gentamicin	8	4/0,5 (S)	8/1 (I)	8	8/1(I)	8/1 (I)
Kanamycin	64	128/2 (I)	8/0,12 (S)	1024	1024/1(I)	1024/1 (I)
Amikacin	32	32/1 (I)	16/0,5 (S)	8	16/2(I)	16/2 (I)
Neomycin	64	32/0.5 (S)	8/0,12 (S)	64	256/4(I)	128/2 (I)
CPZ	64	-	-	512	-	

CPZ -- Chlorpromazine; FIC -- Fractional Inhibitory Concentration; DCL -- Decoct of *Commiphora leptophloeos; *EC -- *Escherichia coli*; S -- Synergism; I -- Indifferent.

**Table 2:** Evaluation of the Modifying Antibiotic Activity of the Decoct of insects from *Anacardium occidentale *(256 μg/mL) and CPZ (16 μg/mL) against aminoglycosides.

	**EC 27**			**EC ATCC8539**		
	**MIC**	**MIC combined**		**MIC**	**MIC combined**	

Antibiotics	-	DAO/FIC	CPZ/FIC	-	DAO/FIC	CPZ/FIC
Gentamicin	8	8/1 (I)	8/1 (I)	8	8/1(I)	8/1 (I)
Kanamycin	64	64/1 (I)	8/0,12 (S)	1024	1024/1(I)	1024/1 (I)
Amikacin	32	32/1 (I)	16/0,5 (S)	8	16/2(I)	16/2 (I)
Neomycin	64	32/0.5 (S)	8/0,12 (S)	64	256/4(I)	128/2 (I)
CPZ	64	-	-	512	-	

CPZ -- Chlorpromazine; FIC -- Fractional Inhibitory Concentration; DAO -- Decoct of *Anacardium Occidentale*; EC -- *Escherichia coli; *S -- Synergism; I -- Indifferent.

Synergism between CPZ and gentamicin was not observed, which is suggestive of the occurrence of another resistance mechanism. Another possibility is a pump that can be affected by the termite decoction from *C. leptophloeos *in the case of gentamicin (Table 1).

## Discussion

Evidence of antimicrobial activity of products isolated from termites has been reported. Peptides such as spinigerin and termicin, isolated from *Pseudocanthotermes spiniger*, showed antifungal and antibacterial activity [22]. Studies on the molecular biology and bioinformatics of the Australian termites of the genus *Nasutitermes *demonstrated their potential as producers of antimicrobial peptides [23,24]. However, as far as we know, no antimicrobial activity of natural products from *N. corniger *in terms of synergism with aminoglycosides or any other antibiotic has been reported so far.

Phenothiazines, such as chlorpromazine, act on the plasma membrane of bacteria affecting efflux pumps and causing alterations in permeability, thereby enhancing the activity of antibiotics, including the aminoglycosides [25-27]. Efflux pumps are known as resistance mechanisms of *E. coli *since the 1980s, belonging to the RND family (resistance nodulation division) and representing a mechanism of multidrug resistance (MDR), which has led to antibiotic resistance to aminoglycosides [28,29].

Animals have been methodically tested by pharmaceutical companies as sources of drugs for modern medical science, and the current number of animal sources for producing essential medicines is quite impressive. The chemical constituents and pharmacological actions of some animal products are already known to some extent, and ethnopharmacological studies focused on animal medicines could be very important in clarifying the eventual therapeutic usefulness of this class of biological remedies [30,31]. As pointed out by Alves and Rosa, further ethnopharmacological studies are necessary to increase our understanding of the links between traditional uses of faunistic resources and conservation biology, public health policies, sustainable management of natural resources and biological prospecting [1].

## Conclusion

The results obtained indicate that decoctions of *N. corniger *(and possibly of other termites) could be a source of natural products with antibiotic modifying activity to be used against multidrug resistant bacteria.

## Competing interests

The authors declare that they have no competing interests.

## Authors' contributions

HDMC carried out the microbial tests; AV review the article and carried out the collection and identification the termite; MAL and GGA-F participated in the design of the study; RRNA conceived of the study, and participated in its design and coordination and helped to draft the manuscript. All authors read and approved the final manuscript.

## Pre-publication history

The pre-publication history for this paper can be accessed here:



## References

[B1] Alves RRN, Rosa IL (2006). From cnidarians to mammals: The use of animals as remedies in fishing communities in NE Brazil. Journal of Ethnopharmacology.

[B2] Alves RRN, Rosa IL, Santana GG (2007). The role of animal-derived remedies as complementary medicine in Brazil. Bioscience.

[B3] Alves RRN, Rosa IL (2007). Zootherapeutic practices among fishing communities in North and Northeast Brazil: A comparison. Journal of Ethnopharmacology.

[B4] Alves RRN, Rosa IL (2007). Zootherapy goes to town: The use of animal-based remedies in urban areas of NE and N Brazil. Journal of Ethnopharmacology.

[B5] Holmgren N (1910). Versuch einer Monographie der amerikanishe *Eutermes *-Arten. Jahr Hamb Wissensch Anstalten.

[B6] Torales GJ, Armua AC (1986). Contribución al conocimiento de las termitas de Argentina (Provincia de Corrientes). *Nasutitermes corniger *(Isoptera: termitidae). Primeira parte. Facena.

[B7] Nickle DA, Collins MS, Quintero DA, Aiello A (1992). The termites of Panama. Insects of Panama and Mesoamerica.

[B8] Constantino R (2002). The pest termites of South America: taxonomy, distribution and status. Journal of Applied Entomology.

[B9] Scheffrahn RH, Krecek J, Szalanski AL, Austin JW (2005). Synonymy of Neotropical Arboreal Termites Nasutitermes corniger and N. costalis (Isoptera: Termitidae: Nasutitermitinae), with Evidence from Morphology, Genetics, and Biogeography. Annals of Entomological Society of America.

[B10] Martius C (1994). Diversity and ecology of termites in Amazonian forest. Pedobiology.

[B11] Fontes LR, Milano S (2003). Termites as an urban problem in South America. Sociobiology.

[B12] Thorne B (1982). Polygyny in termites: multiple prymary queens in colonies of Nasutitermes corniger (Motschulsky) (Isoptera, Termitidae). Insects Sociaux.

[B13] Vasconcellos A, Bandeira AG, Almeida WO, Moura FMS (2008). Termites that build conspicuous nests in two areas of Atlantic Forest under different levels of anthropogenic disturbance. Neotropical Entomology.

[B14] Lu Y, Zhao YP, Wang ZC, Chen SY, Fu CX (2007). Composition and antimicrobial activity of the essential oil of *Actinidia macrosperma *from China. Natural Products Research.

[B15] Mbwambo ZH, Moshi MJ, Masimba PJ, Kapingu MC, Nondo RS (2007). Antimicrobial activity and brine shrimp toxicity of extracts of *Terminalia brownii *roots and stem. BMC Complementary and Alternative Medicine.

[B16] Gibbons S (2004). Anti-staphylococcal plant natural products. Natural Products Reports.

[B17] Coutinho HD, Costa JG, Lima EO, Falcão-Silva VS, Siqueira JP (2009). Herbal therapy associated with antibiotic therapy: potentiation of the antibiotic activity against methicillin--resistant *Staphylococcus aureus *by *Turnera ulmifolia *L. BMC Complementary and Alternative Medicine.

[B18] Coutinho HD, Costa JG, Lima EO, Falcão-Silva VS, Siqueira-Júnior JP (2009). Potentiating effect of *Mentha arvensis *and chlorpromazine in the resistance to aminoglycosides of methicillin-resistant *Staphylococcus aureus*. In Vivo.

[B19] Smith E, Williamson M, Wareham N, Kaatz G, Gibbons S (2007). Antibacterial and modulators of bacterial resistance from the immature cones of *Chamaecyparis lawsoniana*. Phytochemistry.

[B20] Javadpour MM, Juban MM, Lo WC, Bishop SM, Alberty JB, Cowell SM, Becker JM, McLaughlin ML (1996). De novo antimicrobial peptides with low mammalian cell toxicity. Journal of Medicinal Chemictry.

[B21] Mackay ML, Milne K, Gould IM (2000). Comparison of methods for assessing synergic antibiotic interactions. International Journal of Antimicrobial Agents.

[B22] Coutinho HDM, Lôbo KM, Bezerra DAC, Lôbo I (2008). Peptides and Proteins with antimicrobial activity. Indian Journal of Pharmacology.

[B23] Bulmer MS, Crozier RH (2004). Duplication and diversifying selection among termite antifungal peptides. Molecular Biology and Evolution.

[B24] Bulmer MS, Crozier RH (2006). Variation in positive selection in termite GNBPs and Relish. Molecular Biology and Evolution.

[B25] Reddy PH, Burra SS, Murphy PS (1992). Correlation between calmodulin- like protein, phospholipids and growth in glucose-grown *Mycobacterium phlei*. Canadian Journal of Microbiology.

[B26] Salih FA, Kaushik NK, Sharma P, Choudary GV, Murphy PS, Venkitasubramanian TA (1991). Calmoduliln-like activity in mycobacteria. Indian Journal of Biochemistry.

[B27] Kristiansen JE, Amaral L (1997). The potential management of resistant infections with non-antibiotics. Journal of Antimicrobial Chemotherapy.

[B28] McMurry L, Petrucci RE, Levy SB (1980). Active efflux of tetracycline encoded by four genetically different tetracycline resistance determinants in *Escherichia coli*. Procedures of National Academy of Science of USA.

[B29] Van Bambeke F, Glupczynski Y, Plesiat P, Pechere JC, Tulkens PM (2003). Antibiotic efflux pumps in prokaryotic cells: occurrence, impact on resistance and strategies for the future of antimicrobial therapy. Journal of Antimicrobial Chemotherapy.

[B30] Kunin WE, Lawton JH, Gaston KJ (1996). Does biodiversity matter? Evaluating the case for conserving species. Biodiversity: a biology of numbers and differences.

[B31] Pieroni A, Giusti ME, Grazzini A, Fleurentin J, Pelt JM, Mazars G (2002). Animal remedies in the folk medicinal practices of the Lucca and Pistoia Provinces, Central Italy. Des sources du savoir aux médicaments du futur/From the sources of knowledge to the medicines of the future.

